# 846. Pneumocystis Pneumonia in malignancy patients: Who is at risk?

**DOI:** 10.1093/ofid/ofad500.891

**Published:** 2023-11-27

**Authors:** Kritos Vasiloudes, Sadaf Aslam, John Greene

**Affiliations:** Largo Medical Center, Largo, Florida; University of South Florida Morsani College of Medicine, Tampa, Florida; Moffitt Cancer Center, Tampa, FL

## Abstract

**Background:**

Pneumocystis Pneumonia (PCP) is a respiratory pathogen that has been known to cause severe illness in the immunocompromised patients. The pathogen became well known in the HIV epidemic where much of the current standard of practice was first researched. However since the invention of antiretroviral therapy, and advances in cancer therapy the population affected by PCP has shifted to patients with malignancy or a medication induced immunocompromised state. Here we wish to better define the epidemiology and pathogenicity of PCP in the immunocompromised malignancy patients.

**Methods:**

We utilized a retrospective review of 28 consecutive patients from January 1^st^ 2012 to January 1^st^ 2023 that had visited Moffitt Cancer Center with a diagnosis of confirmed or suspected PCP. We collected data on age, underlying malignancy, type of treatment, comorbidities, presenting symptoms, method of diagnosis and computed tomography findings.

**Results:**

Hematologic malignancies comprised 90% of all cases, half of all cases were in patients’ with a type of non-hodgkin’s lymphoma (NHL). The most common malignancy was diffuse large B cell lymphoma 25%, followed by acute myelocytic leukemia treated with stem cell transplant (SCT) 14%, followed by multiple myeloma 11% (Figure 1). The therapy mostly implicated was glucocorticoids in 57% cases, followed by alkylating agents in 32%, and SCT in 25% (Figure 2). The most frequently reported symptoms was dyspnea in 71% of patients, followed by fever 39%, and cough 32%. Diagnosis was made in 21% of patients with serum 1,3-β -D-glucan, 68% were diagnosed following a bronchoalveolar lavage, 11% utilized both (figure 3). of The most common finding on CT was ground glass opacities in 79% of cases. Lymphopenia was present in 79% cases. PCP contributed or was the cause death in 17% cases.

Underlying Malignancy

**Figure:**
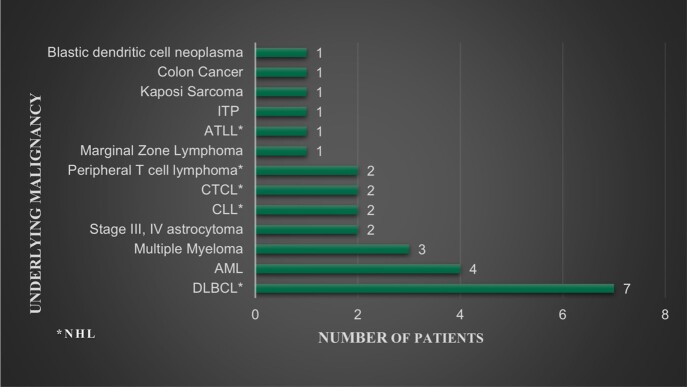

Underlying Malignancy of those who developed PCP pneumonia, n=28 Diffuse large B Cell Lymphoma DLBCL, Acute Myelocytic Leukemia AML, Chronic Lymphocytic Leukemia CLL, Cutaneous T Cell Lymphoma CTCL, Acute T cell Leukemia/Lymphoma ATLL, Immune Thrombocytopenic Purpura (ITP)

Type of cancer treatment

**Figure:**
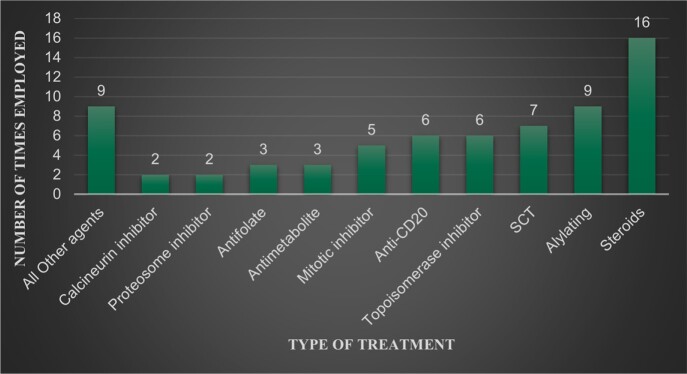

Cancer Treatment of those who developed PCP pneumonia, multiple different therapies were often used in treatment, n=28

Method of Diagnosis of PCP

**Figure:**
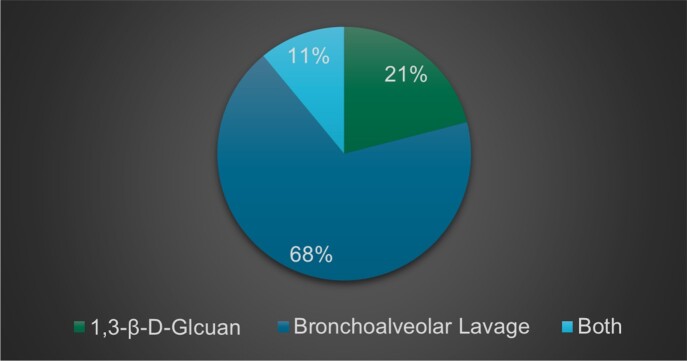

**Conclusion:**

Our data shows that patients’ with NHL comprise half of the patients who developed PCP. Consistent with other studies, steroids were the most frequently implicated therapy, however the second most frequent were alkylating agents. Lymphopenia was commonly associated as well. We wish to bring to light that NHL, Alkylating agents, and particularly lymphopenia as risk factors for development of PCP and should prompt early concern of PCP, particularly those with NHL.

**Disclosures:**

**All Authors**: No reported disclosures

